# Application of Principal Component Analysis in Automatic Localization of Optic Disc and Fovea in Retinal Images

**DOI:** 10.1155/2013/989712

**Published:** 2013-05-23

**Authors:** Asloob Ahmad Mudassar, Saira Butt

**Affiliations:** ^1^Department of Physics and Applied Mathematics, Pakistan Institute of Engineering and Applied Sciences, Nilore, Islamabad 45650, Pakistan; ^2^Isotope Application Division, Pakistan Institute of Nuclear Science and Technology, Nilore, Islamabad 45650, Pakistan

## Abstract

A retinal image has blood vessels, optic disc, fovea, and so forth as the main components of an image. Segmentation of these components has been investigated extensively. Principal component analysis (PCA) is one of the techniques that have been applied to segment the optic disc, but only a limited work has been reported. To our knowledge, fovea segmentation problem has not been reported in the literature using PCA. In this paper, we are presenting the segmentation of optic disc and fovea using PCA. The PCA was trained on optic discs and foveae using ten retinal images and then applied on seventy retinal images with a success rate of 97% in case of optic discs and 94.3% in case of fovea. Conventional algorithms feed one patch at a time from a test retinal image, and the next patch separated by one pixel part is fed. This process is continued till the full image area is covered. This is time consuming. We are suggesting techniques to cut down the processing time with the help of binary vessel tree of a given test image. Results are presented to validate our idea.

## 1. Introduction

This paper presents an extension of the application of principal component analysis (PCA) to retinal images. Localization cases of optic disc and fovea have been presented in the literature [1–16] using techniques other than PCA except the optic disc localization by PCA which is discussed in [2]. In this paper, application of PCA is presented for two different cases: (1) to automatically locate the position of the optic disc in a retinal image, and (2) to automatically locate the position of the fovea in a retinal image. To our knowledge, the latter application is novel and has not been reported in the literature. The former application has been discussed in the literature [2]. The information contained in [2] does not fully appreciate the scope of PCA in optic disc localization. This paper will elaborate the work of optic disc localization and will extend the scope of this work to the localization of fovea. The algorithm we have developed for the localization of optic disc and fovea works faster than the one reported in [2]. The application of PCA to determine the location of the fovea is a relatively difficult problem as compared with locating the optic disc because the fovea usually has lower contrast compared with the optic disc in a retinal image. 

Knowledge of the optic disc location and its diameter is important in the automatic analysis of retinal images. A variety of techniques to automatically determine the location of the optic disc in a retinal image have been described in the literature [1–10]. Most of the techniques do not give satisfactory results. One of the main reasons for the failure of such techniques is that the exudates in a retinal image are sometimes comparable or greater than the size of the optic disc. However, the techniques work very well in a normal retinal image that has no exudates or where the size of the exudates is small enough compared with the size of the optic disc. 

The usefulness of finding the location of an optic disc is that once a point near the centre of an optic disc is determined, then some techniques like the active contour method or other techniques [4, 7] can be used to determine the exact diameter of the optic disc. An increase in the size of the optic disc is an indication of some sort of eye abnormality named peripapillary atrophy. 

Principal component analysis (PCA) is a powerful statistical technique that can be used to identify patterns in data of high dimensions. The technique has applications in face compression and pattern recognition. For pattern recognition problems, some training images are stored in principal component form. Later, this data is used to find the similarity or dissimilarity between an unknown data set and the stored data. Identification of the optic disc and fovea is an example of pattern recognition problems. These two problems have been addressed in this paper using PCA. Application of PCA to retinal images cannot be appreciated without its detailed description which is given in the next section. 

## 2. Mathematical Modeling of PCA with Reference to Retinal Images for Localization of Optic Disc and Fovea

In PCA a set of images is required for training purposes. Let *N* be the total number of images used in the training process with each training image having *n* × *n* dimensions. Training images can be designated by *I*_1_, *I*_2_, *I*_3_,…, *I*_*N*_. These are the intensity images taken from the same part of *N* different retinal images. *I*_*i*_ may represent a patch centred at an optic disc in a retinal image *i* or may represent a patch centred at the fovea in a retinal image *i*. *I*_*i*_ for *i* = 1,2, 3,…, *N* represents *N* patches centred at the optic disc or fovea in *N* different retinal images. Let us define *mn*_*i*_ and *mx*_*i*_ as the minimum and maximum values in the training images as follows:

(1) $$\begin{array}{c} mn_{i}=\mathrm{Min}\left[I_{i}\right],\\ mx_{i}=\mathrm{Max}\left[I_{i}-mn_{i}\right]. \end{array}$$

Normalized training images are obtained using (2):

(2) $$\begin{array}{c} I_{i}^{n}=\frac{I_{i}-mn_{i}}{mx_{i}}255, \end{array}$$

where *I*_*i*_^*n*^ is a normalised version of *I*_*i*_ with intensity in the range from 0 to 255 assuming that the images are grey scale 8-bit images. The mean image of the training images is calculated using the following equation:

(3) $$\begin{array}{c} M=\frac{1}{N}\sum_{i=1}^{N}I_{i}^{n}. \end{array}$$

If we consider the optic discs as training images, then *I*_*i*_^*n*^ would represent either the optic discs taken from the right-eye retinal images or the discs taken from the left-eye retinal images. The right-eye images have optic discs located on the right side in a retinal image, and such images are flipped to align them with the left-retinal images. The mean image *M* is subtracted from each normalised training image *I*_*i*_^*n*^, and the resultant image may be designated with a new symbol *I*_*i*_^*nm*^ and is obtained from the following equation:

(4) $$\begin{array}{c} I_{i}^{nm}=I_{i}^{n}-M. \end{array}$$

The matrix form of *I*_*i*_^*nm*^ can be written as given below:

(5) $$\begin{array}{c} I_{i}^{nm}=\left[\begin{array}{ccccc} I_{i}^{nm}\left[1,1\right] & I_{i}^{nm}\left[1,2\right] & \cdots & \cdots & I_{i}^{nm}\left[1,n\right]\\ I_{i}^{nm}\left[2,1\right] & I_{i}^{nm}\left[2,2\right] & \cdots & \cdots & I_{i}^{nm}\left[2,n\right]\\ \cdots & \cdots & \cdots & \cdots & \cdots \\ \cdots & \cdots & \cdots & \cdots & \cdots \\ I_{i}^{nm}\left[n,1\right] & I_{i}^{nm}\left[n,2\right] & \cdots & \cdots & I_{i}^{nm}\left[n,n\right] \end{array}\right]. \end{array}$$

The matrix elements are flattened to form a set of *n* ^2^ elements, and the flattened list can be designated with the symbol _*f*_ *I*_*i*_^*nm*^:

(6) $$\begin{array}{c} {}_{f}I_{i}^{nm}=\left\{\begin{array}{c} I_{i}^{nm}\left[1,1\right],I_{i}^{nm}\left[1,2\right],\ldots ,I_{i}^{nm}\left[1,n\right],\\ I_{i}^{nm}\left[2,1\right],I_{i}^{nm}\left[2,2\right],\ldots ,I_{i}^{nm}\left[2,n\right],\ldots ,I_{i}^{nm}\left[n,n\right] \end{array}\right\}. \end{array}$$

The required data, *D*, for PCA is formed by combining _*f*_ *I*_*i*_^*nm*^ for *i* = 1,2, 3,…*N* elements as given below:

(7) $$\begin{array}{c} D=\left\{\left\{{}_{f}I_{i}^{nm}\left[k,l\right]\;\forall 1\leq i\leq N\right\},\;\forall 1\leq l\leq n,\;\forall 1\leq k\leq n\right\}. \end{array}$$

The data *D* has a dimension of (*n* ^2^, *N*), and the covariance matrix *C* of *D* has dimension (*N*, *N*) and is given by

(8) $$\begin{array}{c} C=\frac{D^{T}D}{N}. \end{array}$$

A feature vector featvec having dimension (*N*, *N*) is formed by

(9) $$\begin{array}{c} \text{featvec}=\text{Eigen}\;\;\text{value}\left[C\right], \end{array}$$

featvec is used to compute the finaldat_1_ having dimension (*N*, *n* ^2^) and sum_1_ as given by

(10) $$\begin{array}{c} {\text{finatdat}}_{1}=\text{featvec}\cdot D^{T}, \end{array}$$

(11) $$\begin{array}{c} {\text{sum}}_{1}=\sum_{p=1}^{N}\sum_{q=1}^{n^{2}}{\text{finaldat}}_{1}^{2}\left[p,q\right]. \end{array}$$

In words, *D* ^*T*^ has *N* elements each of length *n* ^2^, and each element of *D* ^*T*^ represents one training image. For any number of unknown image patches from a retinal image each having dimension (1, *n* ^2^) to resemble to one of the training images, the error as defined below must have a minimum value for the most similar patch:

(12) $$\begin{array}{c} \text{error}={\text{sum}}_{1}-{\text{sum}}_{2}, \end{array}$$

where sum_2_ has the following definition:

(13) $$\begin{array}{c} {\text{sum}}_{2}=\sum_{p=1}^{N}\sum_{q=1}^{n^{2}}{\text{finaldat}}_{2}^{2}\left[p,q\right], \end{array}$$

finaldat_2_ is found from the following equation:

(14) $$\begin{array}{c} {\text{finatdat}}_{2}={\left(\text{featvec}\left[1\right]\right)}^{T}\cdot \left\{\text{patch}\right\}, \end{array}$$

(featvec[1])^*T*^ has a dimension of (*N*, 1), and each {patch} has a dimension of (1, *n* ^2^), and as a result of matrix multiplication finaldat_2_ is of dimension (*N*, *n* ^2^). 

The mathematical modeling given above explains clearly how the training images are used to determine the similarity between the unknown image and the images in the training set. The training images in our case may consist of either the region centered around the optic disc or the region centered around the fovea. The algorithm that we used in localization of optic disc and fovea follows the steps explained by the mathematical modeling. 

## 3. PCA and Optic Disc Localization

**Figure 1:**
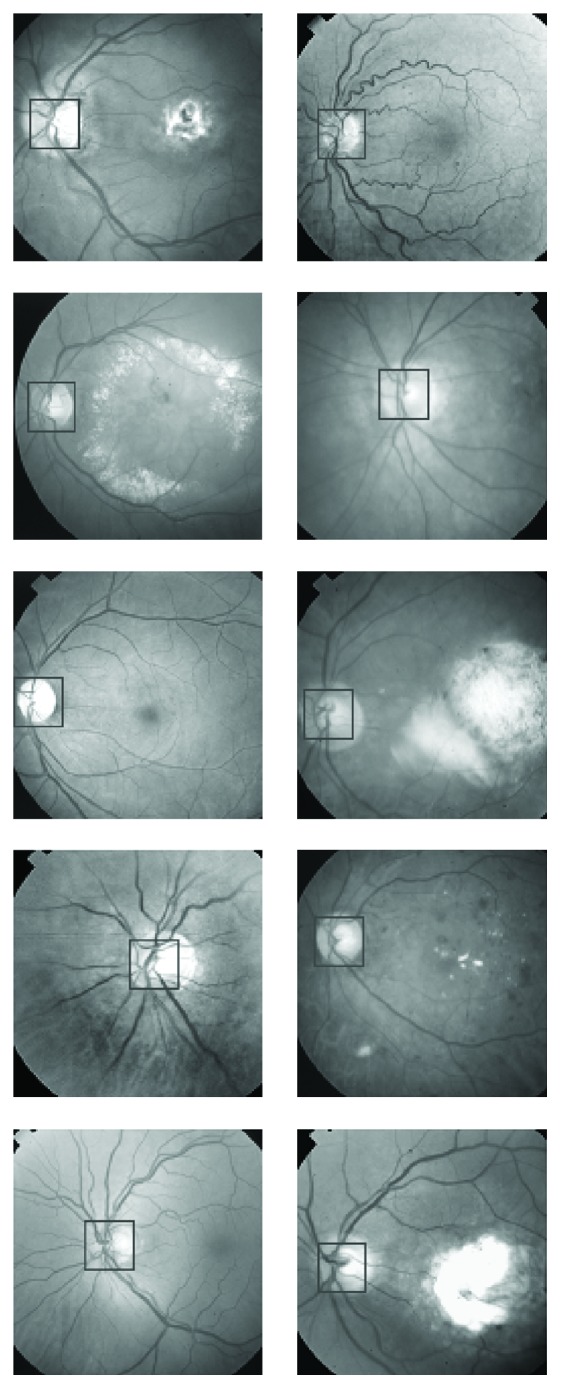
Ten retinal images used as training images in PCA. Areas marked within black squares were used in the training process of PCA.

**Figure 2:**
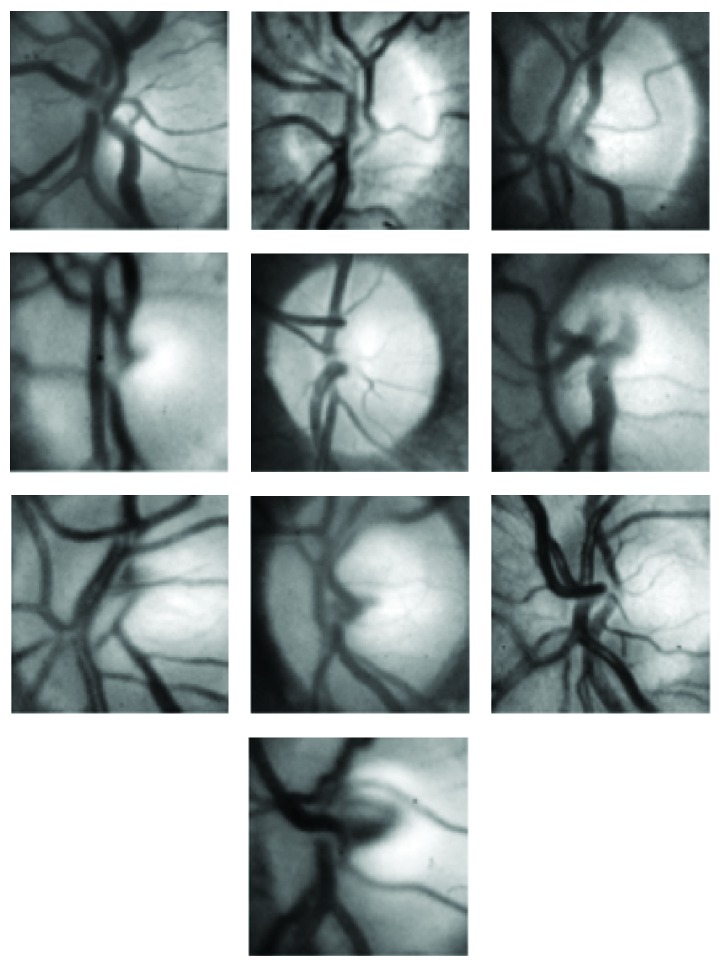
Optic discs indicated in Figure 1 used in the training process of PCA.

The first step in applying PCA to localization of the optic disc is to train the PCA using a set of optic discs from a variety of different retinal images. The training process should cover most of the features of different types and forms of optic discs. For the training purpose, we chose ten retinal images with a variety of optic disc forms. The ten retinal images are shown in Figure 1. The retinal images shown in Figure 1 indicate the position of a square of size 100 × 100 pixels nearly centred at the optic disc covering most of the area surrounded by the optic disc. Some of the images, shown in Figure 1, were right-eye images and were converted to left-eye images using the mirror-image technique. The magnified version of the marked optic discs in Figure 1 is shown in Figure 2. The images shown in Figure 2 were used as training images for the PCA used in localising the optic disc in retinal images. 

**Figure 3:**
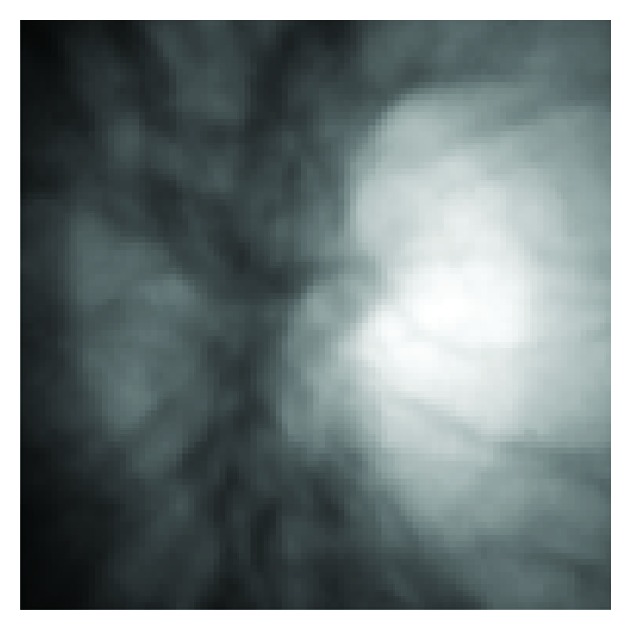
Average image of the ten optic disc images shown in Figure 2.

**Figure 4:**
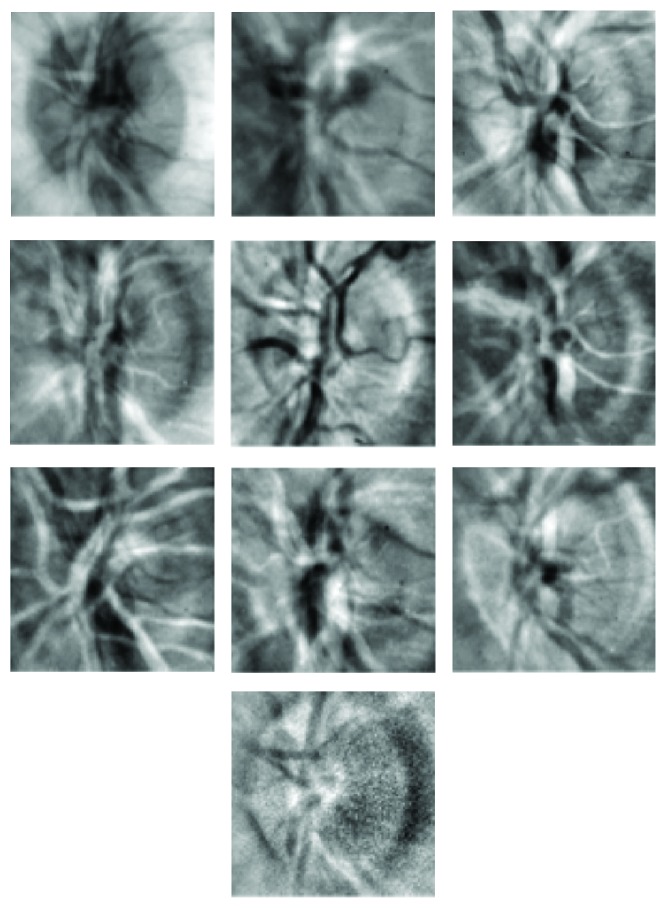
First ten eigendiscs obtained by applying PCA to images in Figure 2.

The training images shown in Figure 2 were normalised in the range 0 to 255 using (1) and (2). These normalised images were then used to compute the average image using (3). The average image so obtained is shown in Figure 3. The average image of Figure 3 was subtracted from each of the images shown in Figure 2 according to (4). The resulting images were then flattened in rows each of length 10000. Ten rows corresponding to ten training images were obtained, and using (6) they were combined to form a matrix of order (10,10000). The transpose of this matrix gives data of dimensions (10000,10). The covariance matrix of the data written in the form of (7) was then calculated using (8). The covariance matrix so obtained had dimensions (10,10). The eigenvalues of the covariance matrix were labelled as featvec having dimensions (10,10). It is the featvec that has the characteristics of all the training images. The featvec is used to compute finaldat_1_ which has dimension (10,10000). The ten elements of finaldat_1_ are called eigendiscs and can be represented as ten images, as shown in Figure 4. The first eigendisc (image at the top left in Figure 4) corresponds to the first principal component, and the tenth eigendisc (image at the bottom right in Figure 4) corresponds to the tenth principal component; sum_1_ is then calculated using (11). 

**Figure 5:**
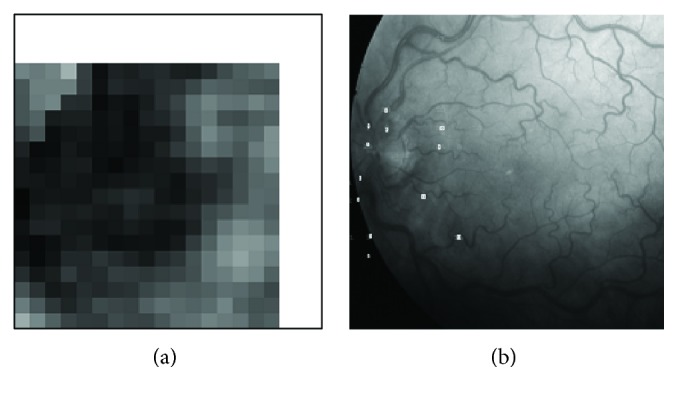
Euclidian distance map (a) of the input image (b). First few minima in the distance map are indicated in (b).

To explain how PCA is used to locate the position of an optic disc in a retinal image, we chose a retinal image as shown in Figure 5(b). The image was split into subimages each of size 100 × 100 called patches. Each patch was substituted in (14), and finaldat_2_ was computed; sum_2_ was then determined using (13), and the error was computed using (12). The error value is then assigned to an area patch in an image which gives the Euclidian distance map and is shown in Figure 5(a). Each patch of size 100 × 100 pixels in the Euclidian distance map represents the value of the error corresponding to the patch at that location in Figure 5(b). Once the Euclidian distance map is formed, the first few minima were computed from the map and are shown in Figure 5(b). A square of size 100 × 100 pixels with its left bottom corner at the minimum position and sides parallel to the image axes is then placed on the input image to mark the location of the optic disc in the input retinal image. 

**Figure 6:**
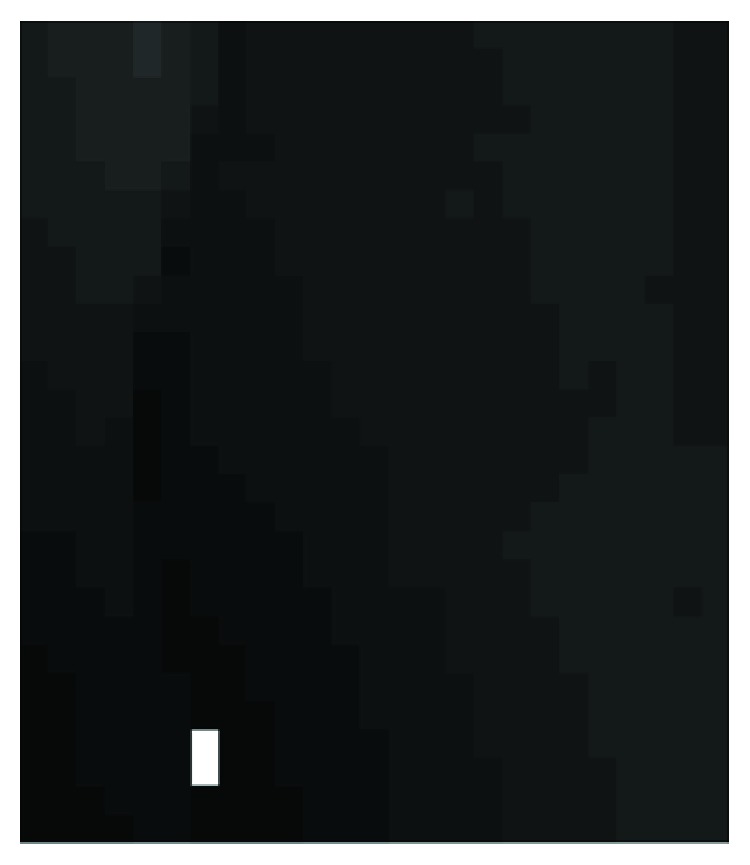
Indication of precise minimum position on the patch with minimum in the Euclidian distance map in Figure 5(a).

The patches in the Euclidian distance map in Figure 5 each has dimension (25 × 25) because the patches of size 100 × 100 pixels with a step size of 25 were extracted from Figure 5(b) and were presented to the PCA. So the exact location of the optic disc is uncertain in an area of 50 × 50 pixels about the minimum position found from the Euclidian distance map. To find an exact location for the optic disc, an area of size 50 × 50 pixels is selected on the input image where the Euclidian distance map gives the minimum, and patches of size 100 × 100 pixels with a step size of 1 are presented to the PCA, and the minimum error then gives the exact location of the optic disc. Such an error image has been shown in Figure 6 where the grey spot precisely locates the position of the optic disc. 

Application of the PCA to the input retinal images saves a lot of computational time when the patches of sizes 100 × 100 pixels with a step size of 25 pixels along both axes are chosen. The simulation work on this problem has revealed that in a very few cases the minimum at the desired location may be missed. To avoid this, the first three minima found from the Euclidian distance map are further explored to find the exact location of the minimum using the techniques described above and shown in Figure 6. It is possible to sort out the candidate regions by clustering the brightest pixels in the input retinal image as mentioned in [2]. To further cut down the computational time, we applied the PCA to the candidate regions only with the strategy described above. This process has significantly reduced the computational time. 

We have also explored a novel idea to cut down the simulation time and to make the PCA effective for retinal images. First, a thick binary vessel tree is extracted from the input retinal image using the matched filtering technique [17]. The PCA was then applied to the input retinal image only at those points of the input retinal image where the binary vessel tree gave unit values. In a binary vessel tree, the vessels are represented by 1 s and the background by 0 s. In this way, only a limited area of the retinal image is explored in the investigation of optic disc localization.

## 4. Optic Disc Localization Results

**Figure 7:**
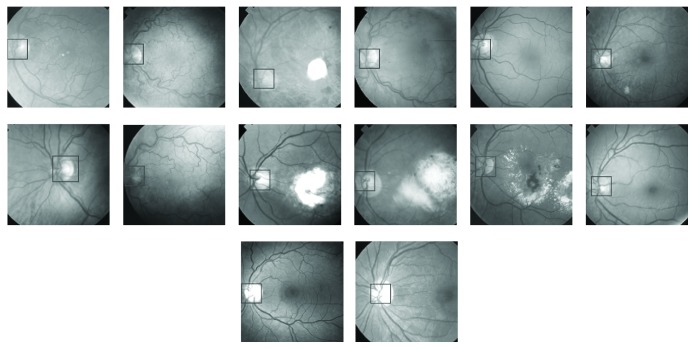
Location of optic discs on the retinal images as determined by PCA.

First, the PCA was trained using 10 optic discs as training images. The training images are shown in Figure 2. PCA was then applied to 50 retinal images out of which only ten images have been shown in Figure 7. The retinal images shown in Figure 7 are the input images to PCA. For each input image in Figure 7, a Euclidian distance map is constructed like the one shown in Figure 5(a). A square boundary of size 100 × 100 pixels is then fitted at the position in the input retinal image corresponding to the minimum position given by the Euclidian distance map. Square boundaries have been drawn on the input retinal images in Figure 7 as determined by PCA. Some input images in Figure 7 are normal retinal images, while others have brighter areas even bigger than the size of the optic disc. The results show that the localization of optic discs in retinal images can be determined sufficiently accurately using PCA. Some of the input retinal images in Figure 7 have faint optic discs with low contrasts where simple techniques, which rely on the brightest region localization as optic discs, cannot work and where the PCA has given good results. The optic discs shown in Figure 2 taken from the input retinal images shown in Figure 1 have got unequal diameters, and the optic discs with unequal diameter were used in training the PCA. Some of the optic discs in the input images shown in Figure 7 also have unequal diameters, but the PCA has worked well in each case. The more the variety in the optic discs used in the training process, the more successful the results obtained from the PCA, and the success rate can be much improved. 

The only drawback with the PCA is that it is computationally very expensive in time as far as the decision on an input retinal image is concerned. The training process is easy and fast. The decision time for an input retinal image can be greatly reduced by taking measures as explained in the previous section. 

## 5. PCA and Fovea Localization

**Figure 8:**
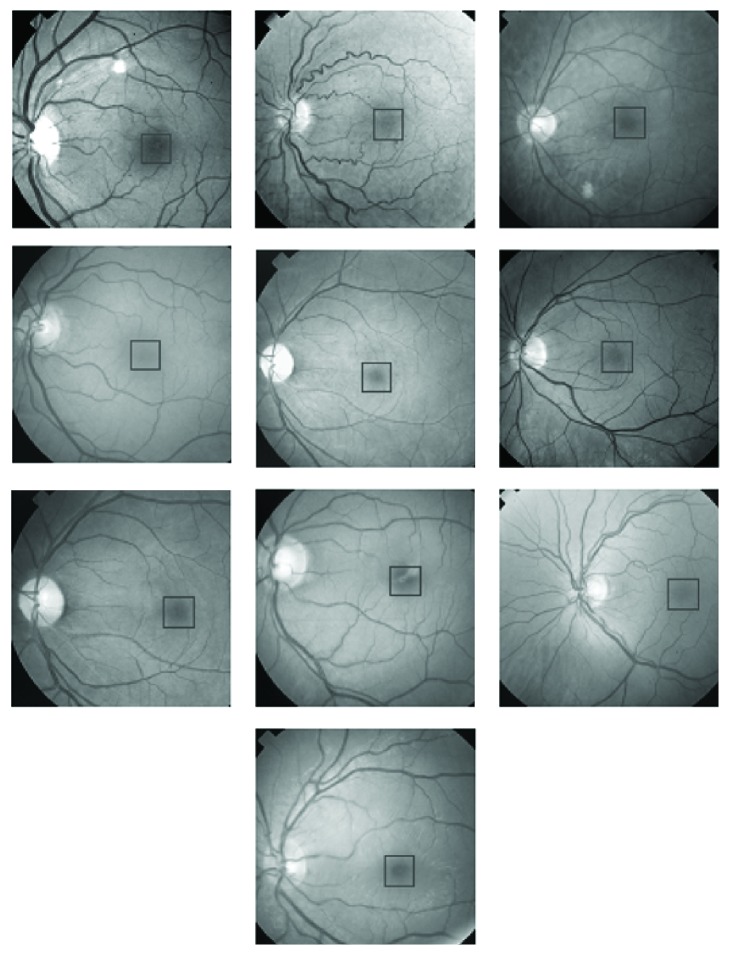
Ten retinal images used as training images in the PCA. Areas marked within black squares were used in the training process of the PCA.

**Figure 9:**
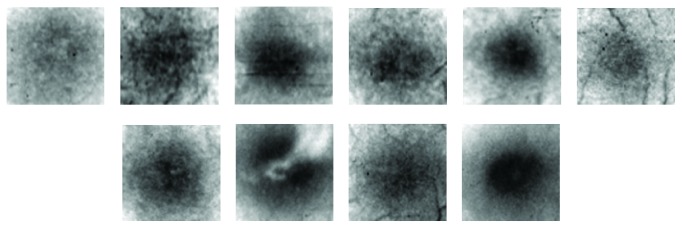
Foveae indicated in Figure 8 used in the training process of PCA.

The first step in the application of PCA to locate foveae in the input retinal images is to choose foveae regions from retinal images for training purposes. Figure 8 shows ten retinal images chosen for training the PCA. Foveae regions have been indicated on the retinal images in Figure 8, and their magnified versions are shown in Figure 9. The foveae regions in Figure 9 are all different from one another in order to introduce versatility into the training process. The size of each fovea in Figure 9 is 70 × 70 pixels. 

**Figure 10:**
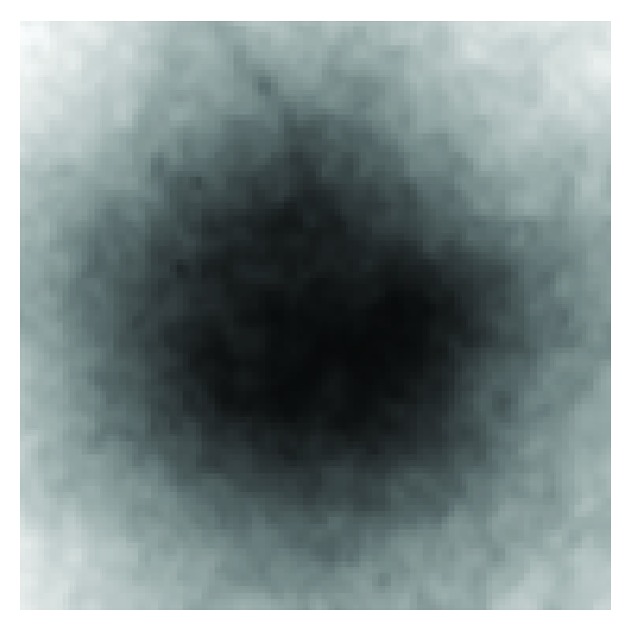
Average image of the ten foveae images shown in Figure 9.

**Figure 11:**
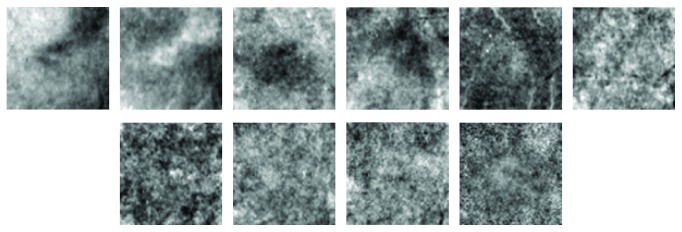
First ten eigenfoveae obtained by applying PCA to images shown in Figure 9.

The fovea images of Figure 9 were normalised using (2), and the average image was then found using (3). The average image is shown in Figure 10. Following the steps given by (4) to (10), finaldat_1_ was computed having dimensions (10,4900). Finaldat_1_ has 10 data sets each of length 4900. These data sets, called eigenfoveae, have been displayed in Figure 11 as ten images each of size 70 × 70 pixels. The final step in the learning process was to compute sum_1_ using (11). 

**Figure 12:**
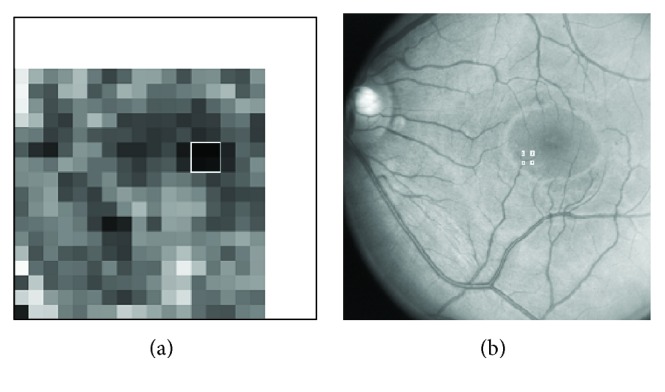
Euclidian distance map (a) of the input image (b). First four minima (contained in a square box with white boundary) in the distance map are indicated in (b).

In Figure 12(b), there is an input retinal image, on which one to four numbers are marked in the region around fovea. The numbers in the region of fovea indicate the first four minima as given by the Euclidian distance map in Figure 12(a). The Euclidian distance map is actually a representation of the error signal computed using (12). 

Sum_2_ varies from patch to patch when the PCA is applied to an input retinal image in Figure 12(b). The first four minima on the input image in Figure 12 represent four patches each of dimension 70 × 70 pixels on the Euclidian distance map. These patches can further be analysed by the PCA with a step size of one to precisely identify the location of the foveae as described earlier and presented in Figure 6. 

**Figure 13:**
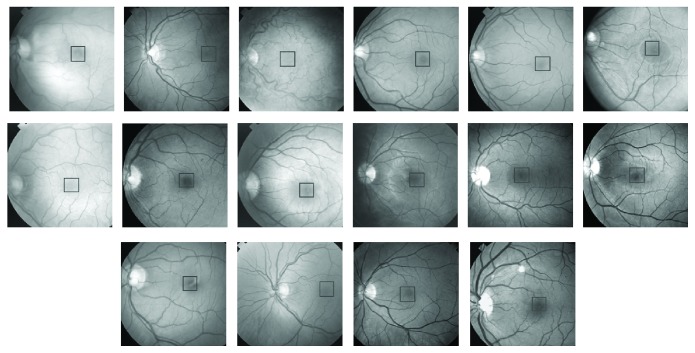
Location of fovea on the retinal images as determined by PCA.

To determine the effectiveness of the PCA in localization of the fovea in retinal images, 70 retinal input images were chosen, and the PCA was tested on them. The success rate was found to be more than 94.3%. A few of the input retinal images on which the PCA was tested have been given in Figure 13. Each of the 20 input retinal images in Figure 13 indicates the location of the fovea as determined by the PCA. Foveae in some of the images in Figure 13 have very poor contrast that even a human eye cannot discriminate properly from the surrounding areas, but the PCA has determined their locations to an outstanding precision. Localization of foveae using the PCA in an input retinal image is more time consuming computationally compared with localization of the optic disc. This is because the PCA can be applied to specified regions when localising the optic disc by choosing candidate regions as clusters of the brightest pixels in a retinal image or by applying the PCA only at thick vessel points using the binary vessel tree of the retinal image. 

The fovea is the darkest region in a retinal image with nearly the same intensity as the blood vessels. The centre of the fovea is usually located from the centre of the optic disc at about 2.5 times the diameter of the optic disc. Even if the location of the optic disc is unknown, the PCA can be applied to the candidate regions, which are clusters of the darkest pixels excluding the blood vessels (using information from a binary vessel tree). This technique then provides a way out to cut down the computation time in locating the fovea by the PCA. The retinal images in Figure 13 have their darkest areas at the edges, which are not part of the retina and can be filled with intensities from adjacent regions. 

## 6. General Discussion on PCA and Related Issues

PCA is generally regarded as a computationally expensive technique which has extremely limited its domain of applications. The size of each retinal image used in fovea localization by PCA was 500 by 500 pixels. The size of the patches from the foveae regions used in training the PCA was 70 by 70 pixels. The same was the size of the patches taken from the retinal images which were presented to PCA for evaluation. The machine on which the computations were done had the following specifications: Pentium(R) D CPU 3.4 GHz, 2 GB of RAM, Microsoft Windows XP Professional Service Pack 2 Version 2002, and Mathematica 4.1 in a stand-alone mode. Foveae from ten retinal images were extracted as pointed out by a human grader and presented to PCA for training. For evaluation, sixteen images were chosen, and patches from one image at a time with a step of 1 pixel along each image axes were selected and fed to PCA, and an automatic square boundary was fit to fovea in each retinal image presented to PCA. The time for this activity excluding the time taken by the human grader in providing the training set of images was 80 hours, 14 minutes, 38 sec, and 109 milliseconds. When the above procedure was repeated with a step size of 25 pixels along each image dimension and the first four minima in the Euclidian distance map were further investigated by taking patches with a 1 pixel step in the region of 4 patches, the measured time was found to be 40 minutes, 13 sec, and 210 milliseconds. The computational time was further reduced by using the vessel tree of each retinal image presented for evaluation as a supporting image. Only those patches from each retinal image were taken for evaluation purposes in which the corresponding patches from the binary vessel trees did not contain any thick vessel. This limited the number of patches used in the evaluation procedure. The time for this activity with a step of 1 pixel was found to be 19 minutes, 9 sec, and 301 milliseconds. This time included the time used to obtain binary vessels tree for each retinal image. The binary vessel tree for each retinal image was obtained using the technique described in [17]. 

The computational time for the application of PCA for the localization of optic nerve head is not available, but we expect a trend in the time measurements similar to that taken by different steps of PCA for fovea localization. The patch sizes were 100 by 100 pixels, and obviously the computational time would be greater in comparison with the time taken by PCA for localization of fovea, where the patch sizes were 70 by 70 pixels, but the size of the retinal images for both activities was 500 by 500 pixels. Binary vessel trees were also used to minimize the number of patches taken from a retinal image for the purpose of evaluation. Only those patches from each retinal image were taken for evaluation purposes in which the corresponding patches from the binary vessel trees contained thick vessel, keeping in view the fact the optic disc is located at the junction of thick vessels. Keeping in view the measured time data available for the application of PCA to fovea localization, we believe that with the help of binary vessels tree we have succeeded in reducing the candidate regions for evaluation purposes, and therefore we have succeeded in reducing the computational time to a reasonably good extent. 

The accuracy of the algorithm was tested on 70 retinal images for foveae and optic discs localization. The size of the optic disc patches was 100 by 100 pixels, and the size of the patches for fovea localization was 70 by 70 pixels. Three human graders were asked to give their best judgment about the centre of optic nerve head and the fovea for each of the images. The accumulated results showed that on the average the results of mean disc centre and the mean fovea centre varied by ±5 pixels leading to a human grader accuracy of 10% and 14.2% for the optic disc and fovea cases, respectively. When 70 retinal images were presented to PCA for evaluation, 68 of the images met the criterion of 10% accuracy leading to an efficiency of 97%, whereas in case of fovea localization, 66 images met the criterion of 14.2% accuracy leading to an efficiency of 94.3%. The 70 retinal images used for the above analysis were normal retinal images with respect to optic disc and fovea. A similar analysis for retinal images corrupted with respect to optic disc and fovea will be dealt in future as the analysis would comprise an extensive and perhaps more difficult work.

The performance of the algorithm was determined on retinal images which can be regarded as normal images with respect to optic disc and fovea. Some of the images in Figure 7 are abnormal images in terms of exudates present in them, but they may be regarded as normal images as far as optic discs are concerned. The two images which failed to fulfill the above-mentioned criterion as far as the optic disc localization is concerned were the images having large exudates present in them, and thick vessels passing through the exudates were prominent resembling much with the original optic discs. But when these two images were presented to PCA in the presence of binary vessel tree, then the problem was resolved. In the absence of assistance from binary tree, the optic disc was located outside the region of actual optic discs, but including the contribution from binary tree the optic discs were found in the designated region, but still the above-mentioned criterion was not met as the exudates peeped into the region close to optic disc covering some of the thick vessels in the candidate regions. These were examples of minor artifact at the optic discs. 

The sixteen images which were presented to PCA for fovea localization are shown in Figure 13. All of the images are normal with respect to fovea. The four abnormal images consisting of large exudates in the candidate regions on which PCA failed to locate foveae at the designated positions are shown in Figure 7. Even the assistance from corresponding binary tree images was useless. A limited number of abnormal images with respect to fovea have been dealt in this research; however, the possible reasons for the failure of PCA in localizing the fovea may be listed as follows: (a) foveal regions may be nonexistent in some retinal images as such regions may be occupied by some eye abnormality, for example, exudates may be covering the candidate regions, (b) the candidate region does exist but with a very poor contrast which may be harder even for a human grader to locate the centre with precision, (c) corneal reflection may mask the foveal region, (d) thin vessels in the vicinity of candidate regions may either be absent or have very poor visibility, in which case the assistance from the binary vessel tree will be useless resulting in the determination of the foveal centers somewhere else in the image, (e) there may be some image artifacts that could prevent the PCA localizing the foveal regions accurately, and the localization may occur far away from the actual region. Future work will address these problems in a greater detail. 

The variability of features in the training set images matters a lot for the PCA to correctly identify the candidate regions with high accuracy. To establish that the variability is important, only two images (first one and the last one) from the second row in Figure 9 were chosen to train the PCA for fovea localization. The small number of training sets implies less variability of features in the training process of PCA. The sixteen images shown in Figure 13 were presented to PCA for fovea localization. It was found that only four images (4th image in the first row from the left, 2nd, 4th, and 6th images from the second row) satisfied the 14.2% accuracy leading to an efficiency of 25%. It may be concluded that variability in the training set of images is an essential part of PCA for high performance in results. Variability in a training set may be improved by combining set members from a variety of different retinal images which at least appear different to a human grader. 

Figure 12(b) has been labeled with first four minima which apparently do not correspond to the centre of the fovea as viewed by a grader. The first four minima were determined from the Euclidian distance map given in Figure 12(a). Some further details are necessary to understand the construction of the Euclidian distance map given in Figure 12 to point out why the first four minima indicated on the retinal image in Figure 12 do not lie close to the fovea centre. The input retinal image in Figure 12 was divided into patches of sizes 70 by 70 pixels with a step of 25 pixels along both the image axes. Each patch from the retinal image shown in Figure 12(b) was presented to PCA, and an error was calculated. This error was displayed as a single grey-level pixel in an empty image called Euclidian distance map at the coordinates corresponding to the lower-left-edge coordinate of the image patch. The number of pixels in the Euclidian distance map along an image axis is equal to the total number of pixels in the retinal image along the same image axes divided by the number of pixels in the step with which the image patches are extracted. The size of the Euclidian distance image is equal to the size of the retinal image divided by the dimensions of the step size. When the Euclidian distance image is displayed in comparative size to the retinal image, the pixeling effect becomes prominent as it is evident from the Euclidian distance map in Figure 12(a). To display the majority of the grey-level pixels in the Euclidian distance map in the lower range for determination of minima, the plot range was set such that the pixels with larger magnitude of errors are displayed white as evident on the right side and on the top side of the Euclidian distance map in Figure 12. 

The four minima indicated on the retinal image in Figure 12 correspond to the first four minima obtained from the Euclidian distance map given in Figure 12. The region around each minimum is further investigated by taking image patches of sizes 70 by 70 pixels in the vicinity of each minimum, and a refined Euclidian distance map is obtained with an accuracy of a single pixel. A square of size 70 by 70 pixels is then drawn on the retinal image with its left-lower edge at the coordinates obtained from the refined Euclidian distance map. Each retinal image given in Figure 13 is, therefore, fitted with a square of size 70 by 70 pixels according to the description mentioned above. 

The images used in training and in the evaluation for both the optic disc and fovea were selected randomly. Figure 1 shows retinal images used in the training of PCA for optic disc localization. Some of the images in Figure 1 are normal images, while others are abnormal or diseased images. The diseased images in Figure 1 show large-sized exudates, but they do not corrupt optic discs. As far as the optic discs are concerned, the images in Figure 1 may be regarded as normal images, and the images for evaluation as in Figure 7 may also be regarded as normal images. The images used in the training (Figure 8) and in the evaluation (Figure 13) of PCA for foveae localization may also be regarded as normal retinal images with respect to foveae. The application of PCA to the diseased retinal images with respect to optic discs and foveae requires separate investigation, and this problem will be dealt separately in future work. 

## 7. Critical Analysis

Segmentation of optic disc in retinal image is well documented [1–16]. Limited work on this subject has been reported using PCA [2]. The segmentation of fovea using PCA is the main novelty of this paper. The segmentation of optic disc using PCA is a relatively simple problem in comparison with the segmentation of fovea using PCA. Fovea has the lowest contrast in a retinal image comparable to the contrast of weak vessels, whereas the contrast of optic disc is generally the highest in a retinal image being the brightest component of the retinal image. It is because the previous attempts for the localization or the segmentation of fovea have been unsuccessful. We have successfully demonstrated the segmentation capability of PCA for fovea. 

There is one major drawback of the PCA, and that is it is a time consuming technique and cannot be applied for real time processing of retinal images for the localization of optic disc and fovea. We have proposed some measures that make PCA work faster especially for the processing of retinal images. In normal process for localization or segmentation of either optic disc or fovea using PCA, test patches from a test retinal image are extracted in a square window which is displaced by one pixel along the horizontal axis or one pixel along the vertical axis for the extraction of the next patch. These patches are fed to PCA for processing which is time consuming. The processing time can be cut down by displacing the window to half of its dimension in either direction, and the patches which are now reduced in number are fed for PCA processing. The patch for which the Euclidian distance map gives the minimum is selected, and a normal procedure of displacing the window by one pixel is applied to refine the localization of optic disc or fovea around that particular patch. 

In a normal retinal image optic disc is the brightest part of a retinal image, and due to this reason its segmentation is simpler and easier than the segmentation of fovea as far as the PCA is concerned. To cut down the processing time further, thick binary vessel tree from the test retinal image is extracted. Optic disc is at the junction of these vessels. Only those test patches are fed to PCA which contain vessels, and this information is obtained from the binary vessel tree of the test image. This reduces the number of test patches for the processing of optic disc localization. At the same time, patches that do not contain the blood vessels are used for the localization of fovea as no vessels pass through this region. This suggests that by selecting candidate regions for the optic disc and fovea, the processing time can be cut down drastically. 

## 8. Conclusion

This paper has described the application of principal component analysis (PCA) to the localization of the optic disc and fovea in a retinal image. Ten optic discs and ten foveae from a variety of retinal images were chosen as training images. The size of the optic disc training images was 100 × 100 pixels and that of the fovea was 70 × 70 pixels. PCA was then applied to 70 retinal images for localization of optic discs and foveae. The two cases were treated separately. The success rate was 97% in the case of optic disc segmentation and 94.3% in case of fovea segmentation. PCA was found to be a promising technique, but it is a computationally time consuming approach. Methods to make the technique faster have been identified separately for both the optic disc and the fovea. The technique cannot be recommended for real time analysis, but it can be utilised in “batch processing” for the localization of optic discs and foveae in retinal images that have been captured previously. 

The PCA technique gives correlation between two objects by comparing Euclidian distance and maximum correlation occuring when the error term is the minimum. The area where the error is the minimum is shown by a square patch. It is assumed that the point lying on the cross-section of two diagonals in that square patch is the most probable centre of the optic disc and the fovea. 

## References

[1] Sinthanayothin C., Boyce J. F., Cook H. L., Williamson T. H. (1999). Automated localisation of the optic disc, fovea, and retinal blood vessels from digital colour fundus images. *British Journal of Ophthalmology*.

[2] Li H., Chutatape O. Automatic location of optic disk in retinal images.

[3] Li H., Chutatape O. (2004). Automated feature extraction in color retinal images by a model based approach. *IEEE Transactions on Biomedical Engineering*.

[4] Li H., Chutatape O. (2003). Boundary detection of optic disk by a modified ASM method. *Pattern Recognition*.

[5] Li H., Chutatape O. A model-based approach for automated feature extraction in fundus images.

[6] Patton N., Aslam T. M., MacGillivray T. (2006). Retinal image analysis: concepts, applications and potential. *Progress in Retinal and Eye Research*.

[7] Li H., Chutatape O. (2003). Automatic detection and boundary estimation of the optic disk in retinal images using a model-based approach. *Journal of Electronic Imaging*.

[8] Niemeijer M., Abràmoff M. D., van Ginneken B. (2009). Fast detection of the optic disc and fovea in color fundus photographs. *Medical Image Analysis*.

[9] Gutiérrez J., Epifanio I., de Ves E., Ferri F. J. An active contour model for the automatic detection of the fovea in fluorescein angiographies.

[10] Sekhar S., Al-Nuaimy W., Nandi A. K. Automated localisation of optic disk and fovea in retinal fundus images.

[11] Zana F., Meunier I., Klein J. C. A region merging algorithm using mathematical morphology: application to macula detection.

[12] Ibañez M. V., Simó A. (1999). Bayesian detection of the fovea in eye fundus angiographies. *Pattern Recognition Letters*.

[13] Chutatape O. (2006). Fundus foveal localization based on vessel model. *Proceedings of the Annual International Conference of the IEEE Engineering in Medicine and Biology Society*.

[14] Estabridis K., de Figueiredo R. J. P. Automatic detection and diagnosis of diabetic retinopathy.

[15] Estabridis K., Defigueiredo R. Fovea and vessel detection via multi-resolution parameter transform.

[16] Pinz A., Bernögger S., Datlinger P., Kruger A. (1998). Mapping the human retina. *IEEE Transactions on Medical Imaging*.

[17] Butt S., Mudasar A. A. Extraction of blood vessels in retinal images using line cross-section of image data.

